# Oncolytic Viruses in Ovarian Cancer: Where Do We Stand? A Narrative Review

**DOI:** 10.3390/pathogens14020140

**Published:** 2025-02-03

**Authors:** Fulvio Borella, Marco Carosso, Maria Pia Chiparo, Domenico Ferraioli, Luca Bertero, Niccolò Gallio, Mario Preti, Jessica Cusato, Giorgio Valabrega, Alberto Revelli, Luca Marozio, Stefano Cosma

**Affiliations:** 1 Gynecology and Obstetrics 1U, Departments of Surgical Sciences, University of Turin, 10126 Turin, Italy; marco.carosso94@gmail.com (M.C.); mariapia.chiparo@unito.it (M.P.C.); luca.marozio@unito.it (L.M.); stefano.cosma@unito.it (S.C.); 2 Department of Gynecology, Léon Bérard, Comprehensive Cancer Centre, 69008 Lyon, France; domenico.ferraioli@lyon.unicancer.fr; 3 Pathology Unit, Department of Medical Sciences, University of Turin, 10126 Turin, Italy; luca.bertero@unito.it; 4 Gynecology and Obstetrics 2U, Departments of Surgical Sciences, University of Turin, 10126 Turin, Italy; niccolo.gallio@edu.unito.it (N.G.); alberto.revelli@unito.it (A.R.); 5 Laboratory of Clinical Pharmacology and Pharmacogenetics, Department of Medical Sciences, University of Turin, 10149 Turin, Italy; jessica.cusato@unito.it; 6 Department of Oncology, University of Turin, Medical Oncology, Ordine Mauriziano Hospital, 10128 Turin, Italy; giorgio.valabrega@unito.it

**Keywords:** ovarian cancer, oncolytic virus, virus, adenovirus, herpes simplex virus, vaccinia virus, myxoma virus, vesicular stomatitis virus

## Abstract

Ovarian cancer (OC) remains the most lethal gynecologic malignancy with limited effective treatment options. Oncolytic viruses (OVs) have emerged as a promising therapeutic approach for cancer treatment, capable of selectively infecting and lysing cancer cells while stimulating anti-tumor immune responses. Preclinical studies have demonstrated significant tumor regression and prolonged survival in OC models using various OVs, such as herpes simplex. Early-phase clinical trials have shown a favorable safety profile, though the impact on patient survival has been modest. Current research focuses on combining OVs with other treatments like immune checkpoint inhibitors to enhance their efficacy. We provide a comprehensive overview of the current understanding and future directions for utilizing OVs in the management of OC.

## 1. Introduction

Epithelial ovarian cancer (EOC) is one of the leading causes of death for cancer, accounting for over 200,000 deaths in 2022 with an incidence of 324,398 new cases worldwide in the same year [1]. EOC constitutes approximately 90% of ovarian tumors and is classified into distinct subgroups, each with unique molecular characteristics, biological behaviors, and clinical features. These subgroups include high-grade serous EOC (the most common histotype), endometrioid, clear cell, mucinous, and low-grade serous EOC [2,3,4,5]. High-grade serous EOC is notable for its numerous molecular abnormalities, particularly the mutation of TP53 in most cases [6,7,8,9,10]. Additionally, somatic and germline mutations in homologous recombination genes, such as BRCA1 and BRCA2, are implicated in the carcinogenesis of EOC [6,7]. This subgroup also shows extensive copy number alterations, involving pathways like FXM1, Rb1, PI3K, and Notch 1 [8,9]. In contrast, clear cell EOC and endometrioid EOC share mutations in genes such as ARID1A, PIK3CA, PTEN, and KRAS [11,12,13]. Mucinous EOCs frequently exhibit KRAS mutations and HER2 amplifications [14,15], while low-grade serous EOC is characterized by the activation of the mitogen-activated protein kinase (MAPK) pathway through mutations in NRAS, KRAS, or BRAF [16,17]. EOC has a poor prognosis, especially among patients diagnosed at advanced stages (International Federation of Gynecology and Obstetrics—FIGO stage III–IV), with a 5-year relapse rate of 75% and low overall survival (OS) [18,19]. In response to these challenges, recent advancements in treatment strategies have emerged. These include optimal debulking surgery to achieve no macroscopic residual tumor and the development of targeted therapies, especially for high-grade serous EOC [19]. One notable advancement is the use of bevacizumab, a drug that targets vascular endothelial growth factor (VEGF), approved for maintenance treatment in high-risk EOC [20,21]. Additionally, poly ADP ribose polymerase inhibitors (PARPi) have been approved for treating EOC, improving progression-free survival (PFS) for BRCA-mutated patients and those with platinum-sensitive relapse [22,23,24,25,26]. Emerging therapies for EOC include immune checkpoint inhibitors (ICIs); however, according to the latest studies, their contribution to survival has proven to be quite limited [27,28,29,30]. This scenario underscores the urgency of investigating alternative treatments that can enhance survival.

The use of oncolytic viruses (OVs) in cancer therapy involves their selective replication in tumor cells, which results in immunogenic cell death and enhances antitumor immunity [31,32,33,34,35]. So far, three OVs have been approved for advanced cancer treatment. Rigvir, an RNA virus derived from the ECHO-7 strain, was approved in 2004 for melanoma treatment [36]. In 2005, China approved H101, a genetically modified adenovirus, for treating nasopharyngeal carcinoma alongside chemotherapy [37]. The US Food and Drug Administration (FDA) approved in 2015 Talimogene laherparepvec (T-VEC), an attenuated Herpes Simplex Virus 1 (HSV-1) encoding granulocyte-macrophage colony-stimulating factor (GM-CSF) for treating unresectable lesions in melanoma patients post-surgery [38]. In this landscape, several studies have also investigated the role of OVs in treating ovarian cancer (OC) [39,40]. This review aims to provide state-of-the-art information on the potential therapeutic role of OVs in EOC.

## 2. Overview of Mechanism of Action

**Figure 1:**
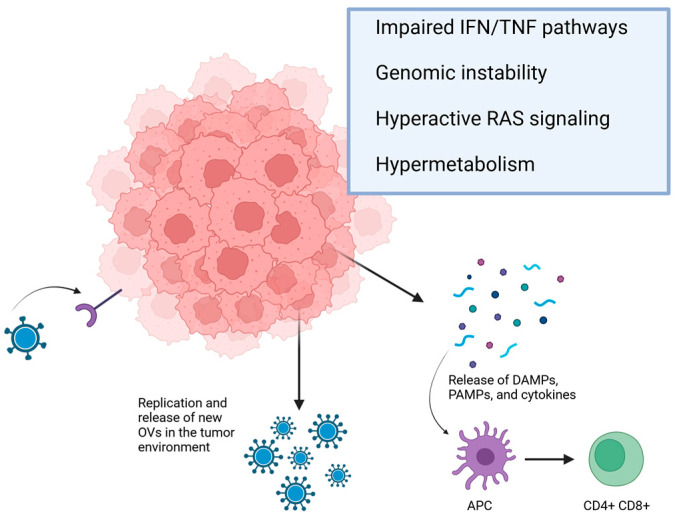
The OVs enter the tumor cell through interaction with specific receptors. The antitumor activity and replication of the OVs are facilitated by tumor cell characteristics such as deregulation of the IFN and TNF pathways, genomic instability, activation of pathways like Ras, and accelerated cell metabolism. The lysed tumor cell releases molecules that activate APCs that trigger the immune response by recruiting CD4+ and CD8+ lymphocytes. Additionally, new OVs are released, infecting other tumor cells. APC: antigen-presenting cells; DAMPs: damage-associated molecular pattern; IFN: interferon; OVs: oncolytic viruses; PAMPs: pathogen-associated molecular patterns; TNF: tumor necrosis factor.

OV therapy is emerging as a highly promising strategy for cancer treatment. These viruses, whether genetically engineered or naturally occurring, are unique in that they selectively replicate within and destroy cancer cells while sparing normal tissues [41]. Unlike gene therapy, which employs viruses as carriers to deliver transgenes, OV therapy uses the virus as an active therapeutic agent [41]. OVs vary greatly in size and complexity, from large, double-stranded DNA viruses like vaccinia (190 kilobases [kb]) and HSV-1 (152 kb), to the much smaller parvovirus H1 (5 kb, linear, single-stranded DNA). These OVs induce cell lysis through different mechanisms during their lifecycle, except for retroviruses, which can be made lytic by expressing toxic transgenes [39]. Most cancer cells have impaired mechanisms for defending against viral infections, such as the interferon (IFN) signaling or tumor necrosis factor (TNF) pathway [42,43,44,45]. Consequently, most viruses can replicate far more efficiently in cancer cells than normal cells. The replication of viruses in the tumor microenvironment leads to the activation of both innate and adaptive immune responses. While this immune activation can limit the spread of the virus, the combination of viral presence and cell lysis, which releases tumor antigens and damage-associated molecular patterns, can help overcome tumor immunosuppression and boost antitumor immunity [46]. The effectiveness of this strategy depends on factors such as pre-existing antiviral and antitumor immunity, as well as the inclusion of immune-stimulatory transgenes. The challenge lies not in making viruses replicate in cancer cells, but in preventing them from replicating in normal cells while maintaining their ability to do so in cancerous ones. The selectivity of OV-based therapy in targeting tumor cells is influenced by several key factors. The entry of the virus into cells via specific, receptor-mediated mechanisms plays a crucial role. Tumor cells in most cases express high levels of viral entry receptors, and efforts are being made to enhance tumor specificity by modifying OVs to target these receptors more effectively [42]. Due to their rapid growth and intense metabolic activity, tumor cells are more conducive to viral replication than normal, non-dividing cells. Moreover, mutations driving tumor growth can further enhance the selectivity of viral replication within cancer cells [42,47,48]. Efforts to ensure cancer cell-specific replication have involved selecting viruses that are naturally non-virulent in humans or genetically engineering virus genomes. For example, Reolysin, a wild-type reovirus (ReoV) variant has oncolytic effects in cells with activated Ras signaling and minimal virulence in normal human cells [49,50]. However, genetic engineering offers greater precision in controlling viral replication. Martuza et al. showed that a genetically modified HSV-1 with a thymidine kinase gene mutation could selectively replicate in cancer cells [51]. This breakthrough paved the way for OVs design and genome engineering advancements. Over the past two decades, a significant development in OV therapy has been the realization that these viruses can induce a robust, systemic tumor-specific immune response during their oncolytic activities [52,53]. This immune response is now acknowledged as a key feature of all OV therapies, potentially improving cancer patient survival [48] significantly. Figure 1 summarizes the main mechanisms of action of OVs.

## 3. Preclinical Studies on Oncolytic Viruses in Ovarian Cancer

Preclinical studies have provided significant insights into the potential of OVs as innovative therapies for OC. These viruses, engineered to infect and kill cancer cells selectively, have demonstrated promising results in laboratory and animal models. Research has focused on various OVs, including adenoviruses, HSV, poxviruses like Vaccinia and Myxoma virus, and others. These studies have explored the mechanisms by which these viruses target OC cells, induce tumor cell lysis, and modulate the immune response. The findings from preclinical models have laid the groundwork for advancing oncolytic virotherapy into clinical trials, highlighting its potential to improve outcomes for patients with OC. This section will review the key preclinical studies, elucidating the therapeutic efficacy and underlying mechanisms of OVs in the context of OC.

### 3.1. Adenovirus

Members of the Adenoviridae family are DNA viruses commonly associated with mild, self-limiting infections in healthy individuals [54]. Among the 57 adenovirus serotypes identified in humans [54], Ad5 is the most widely studied and has been genetically engineered into various conditionally replicative adenoviruses (CRAds) for cancer therapy. CRAds are specifically designed to replicate preferentially in cancer cells. One of the most extensively researched oncolytic CRAds is the E1B 55-kDa gene-deleted Ad5 mutant, dl1520 (also known as Onyx-015, developed by Onyx Pharmaceuticals) [55]. The main mechanism of action of Onyx-015 lies in its ability to infect, replicate, and destroy tumor cells that lack p53 [55,56]. However, recently, some authors studying different serotypes of oncolytic adenoviruses in preclinical OC models have observed different mechanisms via mitochondrial apoptosis and autophagy pathways [57,58], through blocking the cell cycle in the G1 phase and enhancing apoptosis through the PI3K/AKT-caspase-3 signaling pathway [59] or through the activation of JNK/BCL-2/BCL-XL pathway [60].

#### 3.1.1. Immune Response Modulation

The effect of Onyx-015 treatment on OC was tested in both in vitro (cell cultures) and in vivo (mouse models) settings [39,61]. The adenoviral vector expressing IFN-β demonstrated increased cytotoxicity in ovarian carcinoma (OC) cell lines, indicating that the expression of IFN-β enhanced the vector’s capability to target and kill OC cells directly. In a xenograft mouse model, the adenovirus expressing IFN-β significantly reduced tumor growth and improved the survival rates of the mice compared to those treated with unmodified viruses. These therapeutic effects were attributed to direct oncolysis and the activation of the immune system [39,61]. The adenovirus appeared to stimulate the immune response, promoting the infiltration of immune cells, such as natural killer (NK) cells, into the tumor. These immune cells likely contributed to the overall antitumor effects, underscoring the potential of combining oncolytic viruses (OVs) with immune-modulating agents like IFN-β for enhanced cancer treatment [39,61].

Various studies have aimed to enhance oncolytic activity and reduce acute immune stimulation in CRAd therapy by inhibiting the expression of molecules like IL-8 [62], TNF-α [63], or β3 integrin [64]. However, this approach may limit the adaptive anti-tumor immune response [33,65]. Since T cells can target both tumor and viral antigens, the design of CRAds and the route of administration will affect whether an anti-viral or anti-tumor response predominates. Although no significant differences in anti-tumor efficacy were observed between Ad5/3-E2F-Δ24 and Ad5/3-E2F-Δ24-hTNFα-IRES-hIL2 in an immunocompromised model, the cytokine expression enhanced T cell recruitment and activation in an ex vivo EOC model and an immunocompetent model, showing resistance to tumor relapse [66,67,68,69]. Additionally, two oncolytic adenoviruses, Ad5Δ24 and Ad5/3Δ24 armed with GM-CSF, effectively induced tumor- and virus-specific immunity, were well-tolerated and demonstrated clinical benefits in some patients with advanced refractory solid tumors. Specifically, ONCOS-102 (Ad5/3Δ24-GM-CSF) administration induced robust tumor-specific CD8+ T cells both locally in tumors and systemically [70,71,72]. A different approach explored involved the development of an oncolytic adenovirus that incorporates a fusion gene of signal regulatory protein-α (SIRPα) and IgG1 Fc (SG635-SF), which has the potential to inhibit the ‘do not eat me’ signal mediated by CD47 in cancer cells [73]. SG635-SF preferentially promotes the proliferation of hTERT-positive cancer cells, leading to a substantial rise in the SF gene’s presence. The SF fusion protein was reliably detected, and the blockade of CD47 was successfully performed in the SK-OV3 and HO8910 OC cells. In xenograft tissues derived from SK-OV3 cells, there was a significant reduction in CD47 levels and a notable increase in macrophage infiltration, whereas this was not observed in CD47-deficient HepG2 cells, suggesting that the improved antitumor efficacy of SG635-SF relies on CD47. In another investigation, researchers used two types of oncolytic adenoviruses—Ad5 E1A CR2-deletion strain dl922-947 and the chimeric Ad3/Ad11p strain enadenotucirev—to study the role of NK cells in enhancing anti-cancer responses in OC. Given that murine cells do not support the replication of human adenoviruses, the study employed primary human NK cells obtained from both peripheral blood and ascitic fluid of OC patients. Results showed that while dl922-947 and enadenotucirev cannot infect NK cells, they successfully induce their activation and exert anti-cancer cytotoxicity on OC cells that have been infected by adenovirus. Additionally, manipulating NK receptors DNAM-1 and TIGIT significantly affects NK cytotoxicity toward adenovirus-infected cells. These results suggest that NK cells enhance the efficacy of oncolytic adenoviruses in OC, and further boosting NK activity through inhibiting the TIGIT receptor could enhance the therapeutic potential of these viruses [74].

Ad5/3-E2F-d24-aMUC1aCD3-IL-2 (TILT-322) [75] is an armed adenovirus with a human aMUC1aCD3 T cell engager and IL-2. The administration of TILT-322 resulted in heightened T-cell cytotoxic activity, characterized by elevated levels of granzyme B, perforin, and INF-γ. Further analysis revealed that TILT-322 enhanced the activation of γ/δ T cells and influenced various other cell types, including NK cells and NK-like T cells, which are commonly associated with cancer immunotherapy. The TILT-322 therapy also led to a reduction of exhausted CD8+ T cells, as indicated by the expression of immune checkpoints in samples of EOC ascites. The same study group obtained encouraging results with another serotype: 5/3 oncolytic adenovirus encoding a human mucin1 antibody and the human CD3 receptor, Ad5/3-E2F-d24-aMUC1aCD3 (TILT-321) [76]. When TILT-321 was combined with allogeneic T cells, tumor cells were rapidly eliminated. Cells infected with TILT-321 secreted functional aMUC1aCD3, evidenced by enhanced T cell activity and its binding to MUC1 and CD3. In vivo, TILT-321 treatment led to significant antitumor efficacy, driven by increased intratumoral T cell activity in an A549 and patient-derived OC xenograft mouse model humanized with peripheral blood mononuclear cells. A boost in the immune response has been observed in preclinical studies using other serotypes such as Ad5/3-E2F-d24-vIL2 (vIL-2 virus, TILT-452) [77], MEM-288, MUC16-targeting BiTE antibody (OAd-MUC16-BiTE) [78], and Ad5/3-E2F-D24-hTNFa-IRES-hIL2 (TILT-123) [67]. Combined treatment of patient-derived samples with TILT-123 and ICIs targeting PD-1 or PD-L1 in OC cell lines significantly reduced overall cell viability [79]. This combination therapy activated T cells, which showed increased expression of activation markers, and induced beneficial changes in the tumor microenvironment, as evidenced by flow cytometry. Additionally, in an immunocompetent in vivo C57BL/6NHsda mouse model, tumor growth was suppressed with TILT-123, ICIs, or their combination. These findings support the rationale for using TILT-123 virotherapy together with ICIs in OC clinical trials. Other authors reported that combining OVs, CSF-1R inhibitor PLX3397, and anti-PD-1 delayed the progression of ascites, the extent of T-cell infiltration, proliferation, and activation [80].

#### 3.1.2. Coxsackievirus and Adenovirus Receptor (CAR)

A determining factor of the effectiveness of adenoviruses on OC concerns some receptors expressed on cells. The coxsackievirus and adenovirus receptor (CAR) are crucial for adenovirus infection of target cells, influencing infection efficiency. CAR expression levels in OC cell lines (SKOV3, 2774, PA-1, and OVCAR3) correlates with their susceptibility to adenovirus-mediated gene delivery. 2774 and PA-1 cells, which have higher CAR levels on their surfaces, are more susceptible to adenoviral infection compared to SKOV3 and OVCAR3 cells, which have low CAR levels. Transient transfection with the CAR gene significantly increased adenoviral sensitivity in these cells. CAR expression is closely linked to adenovirus infection susceptibility in human OC cells, and enhancing CAR expression can improve the efficiency of adenoviral vector-mediated gene therapy [81]. Unfortunately, CAR and integrins are low-expressed in many epithelial OCs, with these levels being inversely related to the tumor grade [68,82]. Modifying Adenovirus 5 by adding an RGD motif or a polylysine (pk7) motif enhances its binding to integrins or heparan sulfate proteoglycans, respectively. This modification improves gene transfer efficiency into EOC cell lines and primary tumors resistant to Ad5, without affecting the fiber function [83,84,85]. Combining the RGD motif in both the fiber and capsid protein IX significantly increased oncolytic activity in vitro, though it did not show benefits in vivo [86]. Ad5-Δ24RGD demonstrated positive results, completely eradicating intraperitoneal disease in a xenograft mouse model [87]. An alternative strategy to enhance adenoviral tropism through a CAR-independent pathway involves substituting the Ad5 (subgroup C) knob with serotypes from species B and D [88]. After direct injection into tumors, the chimeric Ad5/3 vector, which mainly targets the desmoglein-2 receptor in combination with the CD46 receptor [89,90], demonstrated higher infectivity in EOC cells and subcutaneous tumor xenografts compared to both Ad5 and the RGD variant [91,92]. Additionally, Ad5/3-Δ24 exhibited superior oncolytic effects in cell lines, clinical tumor samples, and an intraperitoneal xenograft murine model compared to Ad5-Δ24RGD, showcasing a favorable safety profile and potential in a phase I clinical trial [93,94,95].

#### 3.1.3. Retinoblastoma (Rb) Pathway

Two additional conditionally replicative adenoviruses (CRAds), dl922–947 and Ad5-Δ24, were engineered with a 24-base pair deletion in the E1A gene, specifically targeting the Rb-binding conserved region 2. This deletion prevents the binding of the E1A protein to the retinoblastoma (Rb) tumor suppressor protein, making these viruses particularly effective in targeting tumor cells with defects in the Rb pathway [96,97]. Normally, the binding of E1A to Rb is essential for the adenovirus to drive S-phase entry and facilitate proper cell cycle progression, which is necessary for optimal viral replication [98]. The Rb pathway is often disrupted in cancer cells, including those in OC, resulting in an abnormal G1-S checkpoint. This disruption allows for Ad5-Δ24 and dl922–947 replication in these tumor cells, as the mutated E1A region can now complement the altered Rb pathway, promoting viral replication [99].

In in vitro studies, both dl922–947 and Ad5-Δ24 exhibited increased cytotoxicity in OC cell lines, such as IGROV1 and OVCAR4, compared to wild-type Ad5 and Onyx-015. This suggests that these CRAds are more efficient in killing OC cells [97,100]. Furthermore, when tested in IGROV1 xenograft models, dl922–947 demonstrated potent anti-tumor activity, comparable to wild-type Ad5, underscoring its potential for OC treatment [100]. Moreover, dl922–947 has shown enhanced antitumor effects in several other cancer xenograft models, surpassing the performance of both Onyx-015 and wild-type Ad5 [97]. These findings highlight the promising therapeutic potential of dl922–947 and Ad5-Δ24, particularly for tumors like OC that often feature disruptions in the Rb pathway, and suggest they may offer superior efficacy compared to other oncolytic adenoviruses.

#### 3.1.4. Smac/DIABLO

A study examined the death mechanisms of three E1A CR2-deleted replicating adenoviruses in OC and the role of the E3 11.6 adenovirus death protein. OC cells were infected with dl922-947 (E3 11.6+) and dlCR2 (E3 11.6−), and a variant, dlCR2 tSmac, also expressed the Smac/DIABLO gene. Traditional apoptosis does not occur, and mitochondria are not involved in adenoviral cell death. Smac/DIABLO expression does not enhance cytotoxicity or apoptotic features. Cathepsin and lysosomal membrane permeability were ruled out, and autophagy, although induced, is not the primary death mode and might support cell survival. There is no evidence of necrosis, and E3 11.6 does not affect the mode or extent of cell death. Thus, the cytotoxicity of E1A CR2-deleted oncolytic adenoviruses in OC could represent a novel form of programmed cell death [101].

#### 3.1.5. Homologous Recombination Deficiency (HRD) Status

In high-grade serous OC, around 15% of patients have germline mutations in BRCA1 or BRCA2. Additionally, The Cancer Genome Atlas (TCGA) consortium data suggests that homologous recombination (HR) defects may be present in 50% of HGSOC cases. These defects can arise from various mechanisms, including somatic mutations in BRCA1/2 and the epigenetic loss of BRCA1 expression [102,103,104]. The impact of HR in the use of OVs was investigated by determining the efficacy of adenovirus type 5 vectors in OC. Research utilizing high-grade serous ovarian cancer cells with matched BRCA2 mutated and wild-type tumors revealed that functional homologous recombination enhances the replication of viral DNA and improves overall effectiveness, while not affecting the processing of viral DNA. These results were corroborated in a larger set of HR-competent and defective OC lines. From a mechanistic perspective, BRCA2 and RAD51 both target viral replication sites inside the nucleus of infected cells, and the positioning of RAD51 is independent of BRCA2. Also, a direct association was noted between RAD51 and the adenovirus E2 protein that interacts with DNA. Finally, functional assays focused on HR efficiency indicate that even though MRE11 is degraded due to Ad5 infection, the ability of cells to repair double-strand breaks in DNA via homologous recombination is not altered. These findings show that Ad5 shifts important HR components to areas where viral replication occurs, boosting cytotoxicity in the process [104,105].

#### 3.1.6. Using Neural Stem Cells to Deliver Adenovirus

The delivery potential of oncolytic adenovirus was investigated using the allogeneic clonal neural stem cell line (HB1.F3.CD21). These NSCs act as a barrier against neutralizing antibodies in the ascitic fluid of patients, ensuring the delivery of oncolytic viral cargo to tumors in preclinical studies of peritoneal OC metastases. CRAd-S-pk7, a type of adenovirus capable of conditional replication, is controlled by the survivin promoter and targets ovarian cancer cells for replication due to their high survivin levels, avoiding replication in healthy differentiated cells. This viral agent, delivered by NSC, has demonstrated its effectiveness against ovarian cancer that is resistant to cisplatin and can be used alongside cisplatin to lessen tumor mass without heightening toxicity levels.

#### 3.1.7. Angiogenesis

EOC development and progression are mainly related to tumor microvessels and the angiogenesis that is modulated by VEGFs, their receptors (VEGFRs), and angiopoietins (Ang) along with their Tie receptors [106]. In this context, the effectiveness of adenoviral gene therapy utilizing soluble VEGFR2 and Tie2, in conjunction with paclitaxel and carboplatin, for addressing OC was evaluated [107]. Gene therapies targeting AdsVEGFR2 and AdsTie2 significantly reduced tumor volume. Moreover, treatment with AdsVEGFR2 and AdsTie2 gene therapy, alone or in combination with chemotherapy, resulted in a notable reduction in ascites accumulation compared to controls. However, VEGF and angiopoietin-2 levels in ascites fluid were elevated following gene therapy. The combined inhibition of VEGF/VEGFR2 and Ang/Tie2 signaling pathways provided an effective therapeutic approach for OC in mice [107]. Other authors evaluated the combination of AAV8-sVEGFR2 and AAV8-sVEGFR3, alongside chemotherapy [108]. This combination effectively diminishes intratumoral angiogenesis and tumor growth in an OVCA mouse model. These results offer preclinical proof-of-concept for utilizing soluble decoy VEGFRs for treating OC.

### 3.2. Vesicular Stomatitis Virus

The vesicular stomatitis virus (VSV) is a highly effective OV. However, its clinical progress has been hindered by two major challenges: significant neurotoxicity observed in rodent and nonhuman primate models, and the rapid production of neutralizing antibodies that inhibit effective repeated systemic use [109,110,111]. However, these limitations can be overcome by pseudotyping VSV with the glycoprotein from the lymphocytic choriomeningitis virus [112,113]. In vitro, a VSV vector expressing GFP (VSV-GFP) demonstrated efficient infection and killing of various OC cell lines and ovarian surface epithelial cells transformed with SV40 T antigen within 3 days [114]. The lack of tumor-specific viral targeting/infection and clearance of the virus by the immune system may compromise the efficacy of VSV-induced oncolysis [114]. In contrast, only about 5% of normal primary human ovarian epithelial cells were infected, and no cytotoxic effects were observed three weeks post-infection [114]. The anti-OC efficacy of VSV-GFP was also evaluated using an immunocompetent white spotting variant (Wv) mouse model. Wv mice have a naturally occurring point mutation in the c-kit gene, leading to defects in germ cell development [115,116].

#### 3.2.1. Immune Response Modulation

VSV-GP exhibited strong oncolytic activity in OC cell lines and xenografts in mice, though remissions were generally temporary. Analyzing the innate immune response showed that OC cell lines could produce type I IFN, eliciting an antiviral state upon viral infection [117]. This response contrasts with other cancer cell lines, which are typically interferon-incompetent. In vitro experiments demonstrated that this antiviral state could be reversed by combining VSV-GP with the JAK1/2 inhibitor ruxolitinib [117]. Ruxolitinib enhances OV treatment in vivo, in both subcutaneous and orthotopic xenograft mouse models, without significant additional toxicity. VSV-GP shows promise as a potent and safe OC treatment, particularly when paired with an interferon response inhibitor [117]. Interestingly, other authors have tested various drugs (PARPi, cytotoxic agents) together with VSV in OC cell cultures and have also observed that JAK inhibitors are more effective because the antiviral interferon pathway was functional in VSV-resistant cell lines [118]. Histone deacetylase inhibitors (HDIs) can modify chromatin epigenetically and blunt the antiviral response of cells. Pretreatment of tumors with HDIs was found to significantly enhance the replication and spread of VSV in OC cells, primary tumor tissue explants, and various animal models. This enhancement is linked to reduced cellular IFN responses and increased virus-induced apoptosis. These findings suggest that HDIs can act as chemical regulators of cellular antiviral responses, promoting the controlled growth of therapeutic viruses in malignancies [119].

#### 3.2.2. VSV Matrix Protein (MP)

Various studies have demonstrated that it is the matrix protein (MP), a structural component of the virion, that causes considerable cytopathogenesis of VSV in the absence of other viral components, including the inhibition of host gene expression, the elicitation of cell rounding and the induction of apoptosis [120]. The antitumor effect and apoptosis-inducing efficacy of a recombinant plasmid encoding VSVMP in human OC both in vitro and in vivo were evaluated. A recombinant plasmid carrying VSVMP-cDNA (VSVMP-p) was created and used to transfect SKOV3 OC cells, which were then analyzed for apoptosis [121]. For the in vivo study, intraperitoneal ovarian carcinomatosis models in nude mice were created and treated with VSVMP-p/liposome complexes, empty plasmid/liposome complexes, liposome alone, or saline solution. Results showed significant apoptosis in SKOV3 cells transfected with VSVMP-p, with a ∼90% reduction in intraperitoneal tumor weight and significantly extended survival in treated mice. Additionally, the antitumor effect was associated with increased NK cell accumulation. The VSVMP-p treatment was well-tolerated without notable toxicity [121]. Similar results have been observed by other authors: administration of VSVMP resulted in significant tumor growth inhibition in xenografts and prolonged the survival of the treated mice. These antitumor effects were associated with marked increases in tumor apoptosis and reductions in intratumoral microvessel density [122]. Other authors observed that MP notably decreased the invasion of VEGFD-SK cells, tumor volume, rates of lymphatic metastasis, and lymphatic vessel density compared to control groups (*p* < 0.05). This was associated with reduced VEGF-D and MMP-2 expression and enhanced apoptosis [123].

### 3.3. Herpes Simplex Virus

Herpes simplex viruses, HSV-1 and HSV-2, are highly prevalent pathogens affecting humans globally, with an estimated prevalence of 67% for HSV-1 and 13% for HSV-2 [124,125]. Both viruses are transmitted through close contact, leading to lifelong infections. HSV-1 is commonly acquired in childhood via the orolabial mucosa, while HSV-2 infections typically occur later in life, predominantly through sexual contact. Infection with one type of HSV generally induces immunity against re-infection with the same serotype, but not against the other [124,125]. Oncolytic HSV are among the select few oncolytic viruses that have progressed to phase III clinical trials [126,127]. One notable advantage is their approximately 30 kb genome, which encodes nonessential genes. This genomic structure allows for extensive genetic manipulation, enabling the addition or replacement of genes through recombination. Additionally, HSV boast a strong safety profile, as they replicate in the nucleus without causing insertional mutagenesis [126]. As regards the specific oncolytic effect, the HSV virus can replicate in cancer cells due to the permissive environment created by the metabolic alterations and abnormal signaling pathways characteristic of tumor cells [128]. Early preclinical studies have demonstrated the efficacy of oncolytic HSV in the treatment of OC; for example, a study explores the efficacy of replication-competent HSV for treating EOC tumors by incorporating membrane fusion capabilities. By using both a syncytial HSV mutant and a hyperfusogenic glycoprotein from gibbon ape leukemia virus, researchers developed Synco-2D, a doubly fusogenic oncolytic HSV. In vitro and in vivo tests showed that Synco-2D significantly increased tumor cell killing and eradicated tumors in 75% of mice with OC xenografts, outperforming conventional oncolytic HSV [129]. Other authors evaluated two attenuated mutant strains of HSV: hrR3, which replaces the gene encoding ribonucleotide reductase with the lacZ reporter gene, and HR522, which induces syncytium formation and expresses the lacZ gene. The efficacy of HR522 was compared with paclitaxel and hrR3 in treating mice with human OC cells. Additionally, the impact of the prodrug ganciclovir (GCV) was examined. The results indicated that high-titer hrR3 significantly prolonged survival compared to paclitaxel, while HR522 combined with GCV markedly enhanced treatment efficacy. This study examined whether chemotherapy resistance affects OC cells’ response to HSV-R3616, an ICP34.5-deficient, replication-restricted HSV-1. Both chemotherapy-sensitive and -resistant EOC cells exhibited similar sensitivity to HSV-R3616 oncolysis. The study also explored the role of p53 in cell death induced by HSV-R3616, finding that loss of p53 function did not impact the sensitivity to HSV-R3616. The results suggest that recombinant HSV-1 lacking ICP34.5 can kill OC cells, including those resistant to chemotherapy and deficient in p53, supporting the potential efficacy of HSV-based oncolytic therapy for chemotherapy-resistant tumors [130]. Promising results have also been achieved using an oncolytic HSV-2 with a deletion in the protein kinase domain of the viral ICP10 gene, which specifically targets the activated Ras signaling pathway in tumor cells [131] or using HSV-1 mutants lacking ICP34.5, capable of killing OC cells that lack p53 function, resist apoptosis, and/or are chemotherapy-resistant [132]. 

#### 3.3.1. Immune Response Modulation

Intratumoral injection of HSV-1716 triggered the expression of IFN-γ, MIG, and IP-10 within the tumor. This led to a significant increase in tumor-associated NK and CD8+ T cells expressing CXCR3 and CD25. Ascites from HSV-1716-treated animals effectively induced the in vitro migration of NK and CD8+ T cells, which was dependent on the presence of MIG and IP-10. Murine monocytes and dendritic cells were responsible for the production of MIG and IP-10 upon HSV-1716 infection. In monocytes, this production was partially inhibited by neutralizing antibodies against IFN-α and -β, indicating a role for type-1 IFNs in this effect. Human OC cells exhibited high numbers of monocytes and dendritic cells. Following HSV-1716 infection, human monocyte-derived dendritic cells produced substantial amounts of IFN-γ and upregulated MIG and IP-10 expression. These findings suggest that HSV-1716 induces an inflammatory response that could enhance antitumor immunity during oncolytic therapy [133]. Furthermore, upon HSV-1716 infection, mouse OC cells showed high levels of viral glycoproteins B and D expression and were highly phagocytized by dendritic cells. Another study [134] aimed to evaluate the anti-tumor activity of an oncolytic HSV engineered to express murine IL-12 and its ability to elicit tumor-specific immune responses. All OC cell lines showed susceptibility to oncolytic HSV in vitro. Mice treated with IL-12-expressing oncolytic HSV exhibited a significantly stronger tumor antigen-specific CD8+ T-cell immune response in the omentum and peritoneal cavity compared to controls. These mice were also more likely to control OC and had significantly longer OS (*p* = 0.02). Notably, five out of six mice treated with IL-12-expressing oncolytic HSV showed no evidence of metastatic tumors at six months, compared to two out of four mice treated with a placebo. Using an HSV amplicon expressing murine GM-CSF combined with HF10 in a murine model, an enhanced immune response was observed. This included an increase in tumor-infiltrating lymphocytes and a rise in splenic CD4+ cells, thereby boosting the immune response against the tumor [135]. Previous studies have shown that VSV-GP may require multiple administrations due to the observed temporary remissions. Conducting studies on immuno-competent mouse models could provide further insights into the efficacy and safety of these vectors in a functioning immune system, better predicting the immune responses in humans [136]. Furthermore, using the Wv model and the QC cell line could facilitate the evaluation of anti-tumor immunity. These models could provide valuable information on anti-tumor immune responses in a context where the immune system is partially compromised but still functional.

#### 3.3.2. Angiogenesis

The capability of replication-restricted HSV-1716, lacking ICP34.5, to infect endothelial cells and disrupt tumor vasculature was investigated [137]. HSV-1716 effectively infected and killed mouse endothelial cell lines H5V and MS1, as well as human umbilical vein endothelial cells in vitro. Capillary tube formation by endothelial cells was inhibited by HSV-1716 both in vitro and in vivo. After intratumoral administration of oncolytic HSV-1716, HSV glycoproteins were detected in CD31-positive tumor vascular endothelium through immunostaining. Viral DNA was recovered from highly purified microdissected tumor vascular endothelium, and treated tumor endothelium showed expression of tissue factor, indicating endothelial damage. Notably, HSV antigen and DNA were also found in endothelium distant from the primary tumor infection sites. Following intravascular inoculation of HSV-1716, viral glycoproteins were associated with tumor endothelium, but not with the vascular endothelium of other organs. Purified tumor endothelial cells demonstrated high proliferative capability and were susceptible to HSV-1716 infection and killing ex vivo, while endothelium from normal organs was not. Additionally, HSV-1716 oncolytic treatment significantly decreased VEGF levels in tumor-bearing animals, thereby dismantling the tumor’s immunosuppressive environment [138].

#### 3.3.3. HER2 Receptor

HER2 protein is overexpressed in 5–19% of EOC, particularly in mucinous EOC [15,139]. By modifying the HSV to include an anti-HER-2 antibody, HSV selectively infects and kills HER-2-positive cancer cells. This engineered virus showed high safety and efficacy in preclinical models (including SK-OV-3 OC cells), significantly inhibiting tumor growth [140]. This strategy demonstrates the potential of retargeting HSVs to specific receptors for targeted cancer therapy, particularly for patients resistant to existing treatments or with tumors that are difficult to reach with conventional therapies [138]. In another study, HSV engineered to target the HER-2 oncoprotein was evaluated in OC and breast cancer cells. The modified virus, R-LM249, specifically infects and kills tumor cells with high levels of human HER-2. R-LM249 was delivered intraperitoneally in mouse models mimicking peritoneal tumor spread. In these models, R-LM249 significantly inhibited peritoneal carcinomatosis, with 60% of treated mice showing no peritoneal spread and a 95% reduction in tumor nodule weight. Additionally, R-LM249 strongly inhibited the growth of ovarian metastases from HER-2+ breast cancer cells and reduced brain metastases [141].

### 3.4. Measles Virus

Measles viruses (MeV) are enveloped pleomorphic particles within the Paramyxoviridae family. They contain one or more copies of a non-segmented, negative-sense RNA genome and exhibit a natural affinity for lymphocytes in humans and primates. Wild-type MeV uses CD150/SLAMF1 as an attachment receptor to enter various immune cells, such as macrophages, dendritic cells, and activated or memory B and T cells. It employs Nectin-4/PVRL4 on airway epithelial cells to exit the host via the respiratory tract [142,143,144]. Natural infection with wild-type MeV causes measles, a disease still responsible for over 100,000 deaths annually worldwide, despite the availability of a safe and effective vaccine [142,143,144].

#### 3.4.1. Immune Response Modulation

In immune-compromised mouse models, the use of a GM-CSF-expressing MeV significantly influenced tumor regression, either hastening or delaying it, which was tied to an elevation in neutrophils from the host and an increase in CD3+ T-lymphocytes within the tumor environment [145,146]. The use of oncolytic MeV, which expresses the murine IFNβ gene, resulted in the recruitment of innate immune cells and a reduction in both tumor growth and angiogenesis in xenograft studies, through the action of immune cells [147]. Additionally, evidence suggests that attenuated expressing Helicobacter pylori neutrophil-activating protein has been shown to effectively induce a significant immune response against tumors in lung and intra-pleural metastatic breast cancer xenografts by enhancing the production of pro-inflammatory cytokines [148]. MeV has been tailored to include other transgenes that enhance immune activity, such as IL-13 [149] inhibitors targeting heat shock proteins [150].

#### 3.4.2. Oncolytic Activity

Recombinant MeV exhibited selective oncolytic activity against OC cells with minimal cytopathic effects on non-transformed ovarian surface epithelium and mesothelium [151]. OC cells expressed high levels of MeV receptor CD46, unlike non-transformed cells. When SKOV3ip.1 human epithelial OC xenografts were subcutaneously injected into athymic mice, the virus led to complete regression in 80% of tumors. Intraperitoneal administration of MeV prolonged the median survival of mice with advanced intraperitoneal SKOV3ip.1 tumor by over 50 days [151]. Similar results were obtained using MeV-CEA with an initially dose-dependent efficacy [152]. The oncolytic efficacy of MeV-CEA can be increased by combining MeV-CEA with thyroidal sodium iodide symporter [153]. Other strategies evaluated to increase the efficacy of MeV were combining it with a yeast cytosine deaminase and uracil phosphoribosyl transferase [154] or delivering it with mesenchymal stem cell carriers to avoid being neutralized by circulating antibodies [155].

### 3.5. Vaccinia Virus

The Vaccinia virus (VACV), a member of the Poxviridae family, has garnered significant attention in the field of oncolytic virotherapy due to its unique ability to selectively infect and lyse cancer cells while sparing normal tissues [156]. Originally employed as a live vaccine for smallpox eradication, VACV has been repurposed as a promising therapeutic agent in cancer treatment. This double-stranded DNA virus has a broad host range and robust replication capabilities, which contribute to its efficacy in oncolytic applications. Additionally, the virus’s large genome accommodates multiple gene insertions, allowing for the engineering of VACV strains with enhanced therapeutic properties [156].

#### 3.5.1. Immune Response Modulation

A preclinical study on the effect of VACV on normal ovaries found that virus-specific CD8 T cells produced both IFN-γ and TNF. Additionally, VACV infection led to the induction of IL-10 and TGF-β, implying that VACV might leverage the ovarian environment to evade the immune system by inducing these suppressive cytokines. Blocking the IL-10 receptor with antagonist antibodies did not enhance VACV-specific CD8 T-cell immunity or virus clearance in the ovaries. VACV replication was only slightly affected in IL-10 knockout mice. Similarly, blocking TGF-β with antagonist antibodies had no impact on CD8 T-cell immunity or VACV replication. Furthermore, an agonist antibody targeting the TNFRSF4 (OX40) receptor increased the number of VACV-specific CD8 T cells producing IFN-γ in lymphoid tissues but did not affect CD8 T-cell infiltration into the ovaries or the viral load [157]. Some authors [158] hypothesized that an oncolytic poxvirus could attract T cells to the tumor site and induce PD-L1 expression in cancer and immune cells, making them more susceptible to anti-PD-L1 immunotherapy. In preclinical models, oncolytic VACV attracts effector T cells and induces PD-L1 expression on both cancer and immune cells within the tumor. This dual therapy approach reduces PD-L1+ cells and promotes efficient tumor infiltration by effector CD8+ and CD4+ T cells, with increased IFN-γ, ICOS, granzyme B, and perforin expression. Additionally, the treatment diminishes the presence of virus-induced PD-L1+ dendritic cells, myeloid-derived suppressor cells, tumor-associated macrophages, and regulatory T cells, as well as double-positive, highly exhausted PD-1+CD8+ T cells. Consequently, this combination therapy may reduce tumor burden and improve survival, potentially extending its applicability to a broader range of cancer patients.

#### 3.5.2. Oncolytic Activity

In preclinical studies, VACV showed high tropism for OC cells [159] and it seems to be particularly effective in chemo-resistant cells [160]. VACV induces necrosis through a marked decrease in intracellular adenosine triphosphate (ATP) levels, disrupted mitochondrial metabolism, and the release of high mobility group box 1 (HMGB1) protein. This necrotic cell death is regulated, as the infection induces the formation of a receptor-interacting protein (RIP1)/caspase-8 complex. Moreover, pharmacological inhibition of RIP1 and its downstream substrates, including mixed lineage kinase domain-like protein (MLKL), significantly reduces cell death. However, blocking TNF-α does not affect the virus’s efficacy, indicating that the necrosis is not a result of autocrine cytokine release [161]. Other strategies to increase the efficacy of VACV that have obtained results in preclinical models consist of armed VACV with a yeast cytosine deaminase gene [162], using trametinib MEK inhibitor [163], cyclooxygenase-2 inhibitors [164] or doxorubicin [165].

### 3.6. Myxoma Virus

The Myxoma virus (MYXV), a member of the Poxviridae family, has emerged as a promising candidate in the field of oncolytic virotherapy. Originally pathogenic to European rabbits, MYXV has shown the ability to selectively infect and lyse human cancer cells while sparing normal tissues [166].

#### 3.6.1. Immune Response Modulation

In vitro studies demonstrated that MYXV treatment enhances the efficacy of cisplatin or gemcitabine, allowing for lower doses than the IC50 for primary OC cells. MYXV also impacts ascites-associated CD14+ myeloid cells from OC patients, reducing their secretion of immunosuppressive cytokines like IL-10 without causing cell death. Pretreatment with replication-competent MYXV, but not replication-defective MYXV, sensitized tumor cells to cisplatin, significantly improving survival in a murine OC dissemination model. This combination therapy enhances the therapeutic benefit of chemotherapy. Furthermore, MYXV followed by cisplatin potentiated splenocyte activation and IFNγ expression, likely by T cells, when splenocytes from treated mice were stimulated with tumor cell antigen ex vivo. The impact on immune responses within the tumor environment underscores the potential of this combinatorial approach [167].

#### 3.6.2. Oncolytic Activity

Activating the PI3K–AKT signaling pathway is crucial for efficient MYXV infection. Inhibition of AKT with Akti-1/2 reduces MYXV infection in monolayer and spheroid EOC cells. Freshly collected ascite infections showed that 54.5% of patient samples were susceptible to MYXV-mediated oncolytic cell death. Additionally, factors in ascites may negatively impact MYXV infection and oncolysis in EOC cells, possibly due to reduced endogenous AKT activity [168].

### 3.7. Reovirus

Mammalian ReoV belongs to the Reoviridae family, which includes non-enveloped RNA viruses with segmented double-stranded RNA (dsRNA) genomes. These reoviruses are found globally and can infect a variety of animals, such as mice, pigs, sheep, bats, and birds. There are four serotypes of mammalian ReoV, labeled as 1, 2, 3, and 4. These serotypes can be differentiated using hemagglutination inhibition or neutralization techniques. In adult humans, ReoV infections are mostly asymptomatic [169]. Nonetheless, recent studies suggest a link between ReoV and the development of chronic conditions like celiac disease [170].

#### 3.7.1. Immune Response Modulation

OC is mostly localized to the peritoneal cavity, making intraperitoneal delivery of ReoV an ideal approach to prevent systemic spread. However, the presence of ascitic fluid in OC can impede oncolytic viral therapy. Without ascitic fluid, ReoV effectively killed OC cells, but this effect was nullified in the presence of ascitic fluid, due to the presence of neutralizing antibodies (NAb) [171]. Using cell carriers like immature dendritic cells, lymphokine-activated killer (LAK) cells, and LAKDC cocultures can enhance virus delivery despite NAb presence. ReoV-loaded LAKDC and iDC were found to shield ReoV from NAb and facilitate tumor cell killing, create a proinflammatory cytokine environment (IFNγ, IL-12, IFNα, and TNFα), and induce an antitumor immune response [171].

#### 3.7.2. Oncolytic Activity

The oncolytic activity of ReoV in OC has been observed in early preclinical studies on cell cultures [172]. Recently, it was observed that oncolytic ReoV induces OC cell apoptosis in a Toll-like receptor 3 (TLR3)-dependent manner [173]. Blocking the chemical interaction between TLR and dsRNA in OV-90 cells and inhibiting the downstream signaling pathways of TLR3 compromised the apoptosis of OV-90 cells caused by reovirus dsRNA. In addition, the infection caused by ReoV leads to a decrease in the levels of cFLIP (cellular FLICE inhibitory protein) within OVCAR3 cells [174]. Down-regulation of cFLIP following treatment of OVCAR3 cells with antisense cFLIP oligonucleotides or PI3K inhibition also increases the susceptibility of OVCAR3 cells to TRAIL-induced apoptosis. When OVCAR3 cells are treated with antisense cFLIP oligonucleotides or subjected to PI3 kinase inhibition, the down-regulation of cFLIP heightens their sensitivity to apoptosis caused by TRAIL. Incorporating cationic liposomes for the delivery of Reo greatly improves its capacity to penetrate OC cells and increases its potency in eliminating these cells in ascitic environments [175].

## 4. Clinical Trials Evaluating Oncolytic Virus for Treating Ovarian Cancer

Several trials have been conducted to investigate the use of OVs in treating EOC (Table 1). Considering the encouraging preclinical results, these studies aim to explore the potential of OVs to selectively infect and destroy cancer cells while stimulating an antitumor immune response.

Vasey et al. completed an early phase I trial assessing intraperitoneal administration of the oncolytic adenovirus dl1520 (ONYX-015) in 16 women with recurrent OC. ONYX-015 selectively replicates in p53-deficient tumor cells. Unfortunately, no tumor responses were observed, but viral DNA persisted for up to 10 days, indicating sustained replication. This trial was the first to use a replication-selective virus in cancer therapy [176]. Ad5-Δ24-GMCSF, an oncolytic adenovirus engineered to express GMCSF, which recruits NK cells and induces tumor-specific T-cell responses, was evaluated in 20 patients with advanced refractory solid tumors (including 4 EOC) [70]. The treatment was well-tolerated and showed a 12.5% complete response rate, with additional disease stabilization. Both injected and non-injected tumors responded, and tumor-specific and virus-specific immunity were demonstrated. Among the evaluable EOC cases, one complete response (CR), one partial response (PR), and one stable disease (SD) were observed. Ad5/3-Δ24-GMCSF combined with low-dose cyclophosphamide to suppress regulatory T cells was also evaluated in another phase I trial [177]. Among 21 patients diagnosed with advanced solid tumors that were resistant to other therapies, the treatment was well-tolerated and showed efficacy in 13 individuals, with 8 out of 12 achieving objective clinical benefits as per RECIST evaluation. A patient with EOC obtained SD. Recombinant adenovirus was evaluated in other phase I trials [95,178]. Overall, the treatment has shown acceptable side effects. Several patients achieved SD but no PR or CR occurred. The safety and efficacy of other recombinant viruses like VACV and pox-virus were evaluated in phase I trials [177,178,179,180,181,182] with results comparable to previous trials evaluating adenoviruses.

In a phase I trial [183], Edmonston vaccine strains of MV-CEA showed antitumor activity in patients with taxol- and platinum-refractory recurrent EOC. Twenty-one patients were treated with MV-CEA intraperitoneally every 4 weeks for up to six cycles. No dose-limiting toxicity or significant adverse effects were observed. Dose-dependent disease stabilization was seen in 14 patients, with a median duration of 92.5 days. Five patients had significant decreases in CA-125 levels. The median survival was 12.15 months, compared to the expected 6 months in this population.

The efficacy of the TroVax^®^ (Modified Vaccinia Ankara, MVA-5T4) versus placebo in extending time to progression in patients with asymptomatic relapsed platinum-resistant EOC, fallopian tube, or primary peritoneal cancer was evaluated by assessing survival and quality of life [184]. TroVax^®^, targeting the 5T4 antigen highly expressed in EOC, has demonstrated safety and efficacy in other solid tumors. The trial focuses on patients with “CA-125 relapse” and low disease volume, for whom early chemotherapy shows no survival benefit. The aim is to delay chemotherapy and evaluate immunological and clinical responses, including delayed effects measured by RECIST and irRC criteria. MVA-5T4 vaccination in patients with asymptomatic relapse was well-tolerated but unfortunately there was no improvement in progression rate at 25 weeks.

Interestingly, a phase II trial evaluated the effectiveness and side effects of paclitaxel with or without wild-type ReoV (Reolysin) in 108 patients with recurrent EOC, fallopian tube, or primary peritoneal cancer [185]. The main goal was to compare PFS between paclitaxel alone and paclitaxel with reovirus. Secondary considerations included OS, response rates, and survival in measurable vs. detectable disease. The median PFS was 4.3 months for the paclitaxel-alone group vs. 4.4 months for paclitaxel p+ reovirus (hazard ratio: 1.11; 90% confidence interval, 0.78–1.59; *p* = 0.687). Unfortunately, the addition of Reolysin to paclitaxel did not improve survival.

The potential role of paclitaxel was also evaluated by adding enadenotucirev in a phase I study involving 38 heavily pretreated platinum-resistant EOC [186]. The study did not report any dose-limiting toxicities; however, the occurrence of frequent catheter-related complications necessitated the cessation of intraperitoneal administration in Phase 1b. Among the patients, 63% reported experiencing at least one treatment-emergent adverse event (TEAE) classified as Grade ≥ 3, with neutropenia occurring most frequently in 21% of cases. Additionally, six patients discontinued their treatment due to adverse effects. After four months, the PFS rate stood at 64%, with a median of 6.2 months. The objective response rate was noted to be 10%, 35% of subjects maintained stable disease, and 65% exhibited a reduction in target lesion burden at one or more assessment points. Among six patients with corresponding pre-treatment and post-treatment biopsies, five showed a marked increase in CD8+ T cell infiltration, averaging a 3.1-fold rise.

Several Phase I trials are currently underway to assess the safety profiles of different OVs. Among these, a noteworthy study is investigating the combination of an engineered adenovirus expressing GMCSF with ICI durvalumab. Additionally, a Phase III trial is evaluating the efficacy of VACV in combination with standard chemotherapy and the angiogenesis inhibitor bevacizumab. Another Phase I trial is assessing the combination of an oncolytic adenovirus coding for TNFα and IL-2 (TILT-123) with pembrolizumab, or pembrolizumab and pegylated liposomal doxorubicin, for the treatment of platinum-resistant or refractory OC. An overview of the ongoing clinical trials is available in Table 2.

**Table 1:** Published results of trials evaluating OVs in the treatment of EOC.

Ref. and Year of Publication	Treatment	Study Design	Clinical Setting	Number of Patients	Clinical Endpoints	Results
Vasey et al., 2002 [177]	Intraperitoneal E1B-55-kd-Gene–Deleted Adenovirus ONYX-015 (dl1520)	Phase I	Recurrent/refractory EOC	16	Feasibility, safety	No evidence of clinical response
Kimball et al., 2010 [179]	CRAd, Ad5-Δ24-RGD	Phase I	Persistent/recurrent EOC	21	Feasibility, safety	15 SD
Cerullo et al., 2010 [70]	Ad5-D24-GMCSF	Phase I	Refractory solid tumors, compassionate use	4	Feasibility, safety	1 CR, 1 SD, 1 PR
Koski et al., 2010 [178]	Ad5/3-D24-GMCSF	Phase I	Refractory solid tumors, compassionate use	4	Feasibility, safety	1 SD
Galanis et al., 2010 [154]	MV-CEA	Phase I	Progressive/recurrent/refractory EOC	21	Objective response	12 SD, median survival 12.5 months
Breitbach et al., 2011 [180]	JX-594 Pox-virus	Phase I	Refractory solid tumors	2	Feasibility, safety	2 SD
Kim et al.2013 [95]	Intraperitoneal Ad5/3-Δ24	Phase I	Recurrent EOC	10	Feasibility, safety	6 SD, 2 PD
Cohn et al., 2017 [186]	Reolysin + paclitaxel vs. paclitaxel alone	Phase IIB	Recurrent or Persistent Ovarian Epithelial, Fallopian Tube, or Primary Peritoneal Cancer	108	PFS	Median PFS: 4.3 months for paclitaxel; 4.4 months for paclitaxel + reovirus
Lauer et al., 2018 [181]	Vaccinia virus GL-ONC1	Phase I	Advanced peritoneal carcinomatosis	3	Feasibility, safety	2 PD, 1 NA
Holloway et al., 2018 [182]	VACV GL-ONC1	Phase I	Heavy pretreated platinum-refractory/resistant EOC	11	Safety, survival	DCR (PR + SD): 55%
Moreno et al., 2021 [187]	Enadenotucirev + paclitaxel	Phase I	Recurrent pretreated platinum-resistant EOC	38	PFS	4-month PFS rate: 64% (median 6.2 months); ORR: 10%
Pakola et al., 2024 [183]	TILT-123 adenovirus	Phase I	Refractory solid tumors	3	Safety, tumor response	NA
Michael et al., 2024 [185]	MVA-5T4 vs. placebo	Phase II	Relapsed EOC	94	PFS	3 months with MVA-5T4; 3 months with placebo

EOC: epithelial ovarian cancer; DCR: disease control rate; GMCSF: granulocyte-macrophage colony-stimulating factor; MVA: modified vaccinia ankara; MV-CEA: measles virus—carcinoembryonic antigen; NA: not available; PD: progression disease; PR: partial response; SD: stable disease; VACV: vaccinia virus.

**Table 2:** Ongoing trials on the use of OVs in the treatment of EOC.

Trial	Study Start	Treatment	Study Design	Clinical Setting	Enrollment	Clinical Endpoints
NCT02068794	2014 April 25	MV-NIS infected mesenchymal stem cells	Phase I/II	Recurrent EOC	34	Safety/mean 12-months OS
NCT02364713	13 March 2015	MV-NIS vs. standard chemotherapy	Phase II	Platinum-resistant EOC	66	PFS
NCT02759588	May 2016	VACV GL-ONC1	Phase I/II	Recurrent or Refractory EOC	46	Safety, PFS
NCT02963831	7 September 2017	ONCOS-102; Ad5/3-D24-GMCSF + durvalumab	Phase I/II	Advanced peritoneal malignancies	67	ORR
NCT03225989	1 March 2018	LOAd703	Phase I/II	Advanced cancers (including EOC)	47	Safety, PFS, OS
NCT05180851	30 November 2021	L-IFN adenovirus	Phase I	Relapsed/refractory solid cancers (including EOC)	28	Safety
NCT05271318	17 May 2022	Adenovirus (TILT-123) with pembrolizumab or pembrolizumab and pegylated liposomal doxorubicin	Phase I	Platinum-resistant or refractory EOC	29	Safety
NCT05281471	31 August 2022	VACV + chemotherapy + bevacizumab	Phase III	Platinum-resistant/Refractory EOC	186	PFS
NCT05801783	2 December 2022	HSV-1 R130	Phase I	Relapsed/refractory EOC	10	Safety
NCT05684731	1 February 2023	VACV K1	Phase I	Recurrent or refractory EOC	30	Safety, efficacy
NCT06508307	26 April 2023	VACV GC001	Phase I	Advanced solid tumors (including EOC)	21	Safety

EOC: epithelial ovarian cancer; GMCSF: granulocyte-macrophage colony-stimulating factor; HSV: herpes simplex virus; DCR: disease control rate; MV-NIS: measles virus encoding the human thyroidal sodium iodide symporter; ORR: objective response rate; OS: overall survival; PFS: progression-free survival, VACV: vaccinia virus.

## 5. Discussion and Conclusions

OC remains one of the most lethal gynecologic malignancies, characterized by a high mortality rate and limited effective treatment options. Despite advances in surgery and chemotherapy, the prognosis for OC patients, especially those with advanced-stage disease, has seen little improvement over the years [187]. Consequently, there is an urgent need for innovative therapeutic strategies to enhance survival outcomes.

OVs have emerged as a promising class of therapeutic agents in cancer treatment. These viruses selectively infect and lyse cancer cells while stimulating an anti-tumor immune response. Their unique mechanism of action offers the potential to overcome resistance to conventional therapies and provides a novel avenue for targeting OC.

Preclinical studies have demonstrated the efficacy of OVs in OC models. Utilizing cell cultures alongside xenograft models achieved significant decreases in tumor growth and longer survival times. Among the various OVs, HSV and adenovirus have been the most extensively studied in OC. HSV-based therapies have shown potent oncolytic activity and the ability to induce robust immune responses. Similarly, adenovirus has been engineered to selectively replicate in and destroy cancer cells, showing promising preclinical results.

Clinical trials of OVs in OC have predominantly been early-phase studies (Phase I), aimed at evaluating safety and tolerability. These trials have generally reported a favorable safety profile, with manageable side effects and minimal toxicity. However, the impact on patient survival has been modest, indicating that while OVs are safe, their therapeutic efficacy in the clinical setting remains to be fully established.

Ongoing Phase I trials explore combining OVs with immunotherapies, such as ICIs, to enhance their therapeutic potential. These combination strategies aim to leverage the immune-activating properties of OVs to augment anti-tumor immune responses and overcome OC’s characteristic immunosuppressive tumor microenvironment.

Several factors can account for the limited success of OVs in clinical settings [175,188,189,190,191,192]. One potential mechanism is the presence of the tumor microenvironment, which can inhibit viral replication and spread. Additionally, pre-existing immunity to the viral vector can neutralize the virus before it exerts its oncolytic effects. Moreover, the heterogeneity of OCs may necessitate personalized approaches to optimize OV therapy.

Another critical factor to consider is the delivery method of OVs. Intratumoral injection, while effective for accessible tumors, may not be practical for metastatic or deeply seated tumors. On the other hand, systemic delivery faces challenges such as rapid clearance by the immune system and non-specific uptake by non-tumor tissues. Advances in viral engineering and delivery methods, such as encapsulating viruses in nanoparticles or using targeted delivery systems (e.g., stem cells), may help overcome these challenges and improve the therapeutic efficacy of OVs.

Moreover, the immunosuppressive tumor microenvironment in OC poses a significant barrier to the success of OVs. Tumor-associated macrophages, regulatory T cells, and myeloid-derived suppressor cells can inhibit the anti-tumor immune response induced by OVs. Strategies to modulate the tumor microenvironment and enhance the immune response, such as combining OVs with ICIs or cytokine therapies, are being explored and hold promise for improving outcomes.

In conclusion, while OVs represent a promising and innovative approach to OC treatment in preclinical studies, their clinical efficacy has yet to be fully realized. The safety profile observed in early-phase trials is encouraging, and ongoing research into combination therapies holds potential for improving outcomes. Future studies should focus on elucidating the resistance mechanisms and optimizing the delivery and targeting of OVs. Through continued investigation and clinical development, OVs may become vital to the therapeutic arsenal against OC.
